# Differential alterations of lateral entorhinal cortex impact on CA1 pyramidal neuron subpopulations in PS19 mouse model of tauopathy

**DOI:** 10.1002/alz.087085

**Published:** 2025-01-03

**Authors:** Chengju Tian, Isabel Reyes, Mohankumar Thangavel, Arjun V. Masurkar

**Affiliations:** ^1^ New York University Grossman School of Medicine, New York, NY USA; ^2^ NYU Grossman School of Medicine, New York, NY USA

## Abstract

**Background:**

The lateral entorhinal cortex (LEC), followed by area CA1 of hippocampus, are interconnected brain areas implicated early in Alzheimer’s disease (AD). Processing of LEC input by CA1 pyramidal neurons (PNs) is critical for non‐spatial memory, in which deficits are seen in early AD. How this process is affected by tauopathy is unclear. Furthermore, CA1 PNs are divided into molecularly and functionally distinct superficial (sPN) and deep (dPN) subpopulations. It is additionally unclear if and how LEC input processing by these subpopulations are differentially impacted.

**Method:**

LEC of female PS19 and WT mice (8‐10 month old) were injected with an AAV expressing channelrhodopsin ChrR2. After two weeks, acute hippocampal slices were prepared and whole cell patch clamp recordings were performed on dPNs/sPNs of distal CA1 in current clamp and voltage clamp mode using a potassium‐ or cesium‐based intracellular solution, respectively. Synaptic responses were measured in current clamp and voltage clamp mode, with LEC axons optogenetically activated by blue LED light. In current clamp, excitatory postsynaptic potentials (EPSPs) were measured with inhibition blocked. In voltage clamp, excitatory postsynaptic currents (EPSCs) were evaluated by holding membrane potential at ‐70mV, and inhibitory postsynaptic currents (IPSCs) were evaluated by holding at 0mV.

**Result:**

All data is n = 3‐8/group. Compared to WT, LEC‐driven EPSCs showed significant reductions in PS19 sPNs (18.7pA vs. 8.4pA, p = 0.0016) but were unchanged in dPNs (12.4pA vs. 13.6pA, p = 0.2841). Measurements of LEC‐driven EPSPs showed analogous findings. There was no change in paired pulse ratio, ruling out presynaptic changes. There was no change in EPSP decay time constant or temporal summation of the EPSP, ruling out changes in postsynaptic excitability. In contrast to excitation, LEC‐driven IPSCs were unchanged in PS19 versus WT mice in both sPNs (45.7pA vs. 51.9pA, p = 0.62) and dPNs (25.46pA vs. 22.3pA, p = 0.22).

**Conclusion:**

LEC drive of CA1 PNs exhibits a cell type‐specific vulnerability to tauopathy, with sPNs differentially susceptible to a reduction of excitation, without changes in feedforward inhibition. Our results further support a postsynaptic mechanism that may be due to spine loss, changes in spine morphology, or shifts in glutamate receptor expression.

